# A Leadership and Managerial Competency Framework for Public Hospital Managers in Vietnam

**DOI:** 10.3934/publichealth.2017.4.418

**Published:** 2017-08-16

**Authors:** Phan Van Tuong, Nguyen Duc Thanh

**Affiliations:** Department of Hospital Management, Health Management Training Institute, Hanoi University of Public Health, 1A Duc Thang Road, Duc Thang Ward, North Tu Liem district, Hanoi, Vietnam

**Keywords:** Leadership and managerial competency, competency framework, hospital managers

## Abstract

**Objective:**

The aim of this paper was to develop a leadership and managerial competency framework for public hospital managers in Vietnam.

**Methods:**

This mixed-method study used a four-step approach. The first step was a position description content analysis to identify the tasks hospital managers are required to carry out. The resulting data were used to identify the leadership and managerial competency factors and items in the second step. In the third step, a workshop was organized to reach consensus about the validity of these competency factors and items. Finally, a quantitative survey was conducted across a sample of 891 hospital managers who are working in the selected hospitals in seven geographical regions in Vietnam to validate the competency scales using exploratory factor analysis (EFA) and Cronbach's alpha.

**Results:**

The study identified a number of tasks required for public hospital managers and confirmed the competencies for implementing these tasks effectively. Four dimensions with 14 components and 81 items of leadership and managerial competencies were identified. These components exhibited 83.8% of variance and Cronbach's alpha were at good level of 0.9.

**Conclusions:**

These competencies are required for public hospital managers which provide guidance to the further development of the competency-based training for the current management taskforce and preparing future hospital managers.

## 1. Introduction

In Vietnam, since the early 1990s, there had been profound changes in health system such as health insurance reform, decentralization and autonomy in health facility management and a rapid development of the private health system [1],[2]. These changes have required hospitals to improve performance and demonstrate greater transparency and accountability in the context of the increasing health care needs [3],[4].

Leadership and managerial competencies play a very important role in achieving effectiveness and efficiency of health facilities' performance in low and middle income countries. Hospital managers should have sufficient levels of leadership and managerial competencies to co-ordinate the complex environment. In Vietnam, however, little attention has been paid so far to management training. The current management training programs have not kept up with the changes in health policies [5].

Competency is defined as a person's ability to perform his/her job [6]. Competency consists of knowledge, attitudes and skills required to implement a task or job [7]–[9]. The Central Committee of the Communist Party of Vietnam has recently issued Resolution No 29-NQ/TW which emphasizes that a competency-based education and training framework be adopted for health professionals, including hospital managers [10]. In addition, the Ministry of Health has been developing the regulations and directives required in the promotion of health professionals to the management positions [11]. One of the requirements for promotion is that health service managers must have undertaken hospital management training.

Globally, many competency-based training programs have been developed to meet the training needs of hospital managers [6],[9],[12],[13]. In Vietnam, however, similar training programs have not been developed because very few studies have examined the required leadership and managerial competencies for hospital managers. The reason for this is that hospital management has not been considered to be important [5]. The fact is that hospital managers have focussed on their clinical profession where they can easily forsee and earn the pecuniary benefits. As a consequence, they have paid little attention to the leadership and managerial side of the profession where these benefits are not as immediate. This is a misconception because the leadership and managerial competencies will help hospital managers to better manage their hospital's resources and assist them to prevent and deal with the problems that may adversely impact on their hospitals. In light of this, it is important to identify the relevant leadership and managerial competencies which are recognized by the profession and regarded as essential to a well-managed public hospital system in Vietnam. These competencies will be used not only to orient the training curriculum development to meet the requirements of the above regulations and directives, but also to select and recruit appropriate candidates to the management positions.

In literature, several different approaches have been used to identify the core leadership and managerial competencies for health professionals. Mixed methods were used in identifying the competencies for hosptial managers and community health service managers [6],[12]. Other studies applied merely qualitative methods to identify the required leadership and managerial competencies for hospital managers [14]–[16]. These studies are limited in that the identified competencies were not confirmed by its validity and reliability through statistical techniques. Few studies dealt with that issue [9],[17].

This paper aims to develop a leadership and managerial competency framework for public hospital managers in Vietnam. This framework will be used as a basis to develop the appropriate training curriculum for hospital managers in Vietnam.

## 2. Methods

This exploratory and mixed-method study used a four-step approach to collect both qualitative and quantitative data via document analysis, competency identification, workshop to validate identified competencies and quantitative survey for verification.

**Figure 1.**
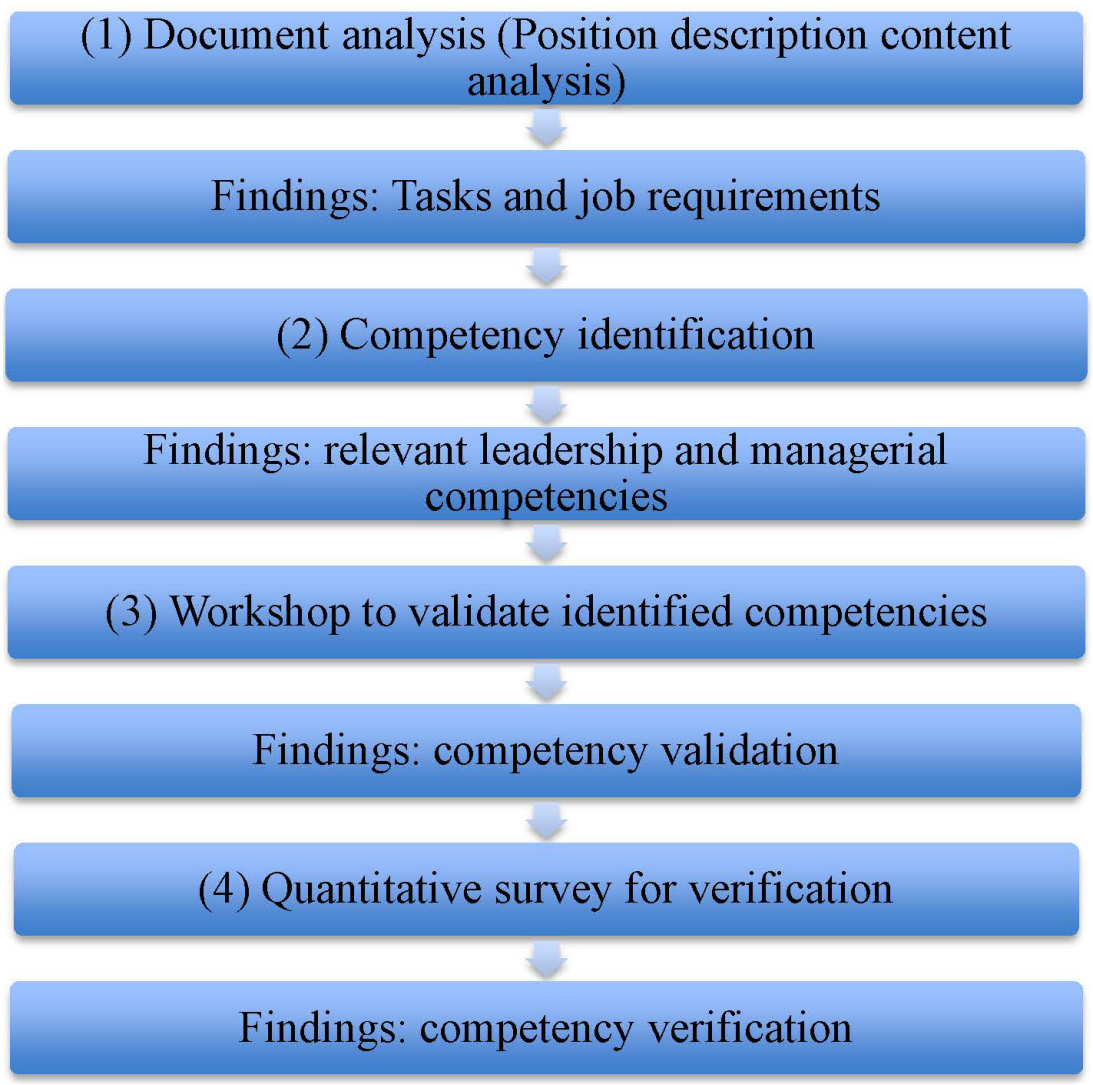
The steps in developing leadership and managerial competency.

It can be seen from Figure 1 that the first step was a position description content analysis through document analysis to identify the tasks and job requirements public hospital managers are required to carry out. These tasks were clearly written in a hospital regulation document issued by the Ministry of Health [18]. In the second step, the resulting data were used to identify the leadership and managerial competency factors needed to perform these tasks effectively. In the third step, a workshop was organized to reach agreement about validity of these competency factors. Finally, in the fourth step, the quantitative survey was conducted to collect data to rate the degree of importance of the leadership and managerial competencies.

### 2.1. Research subjects

For the workshop, research subjects included health professionals who are heads of divisions from the Ministry of health and the provincial Department of health, as well as heads of health and hospital management departments from the medical universities and medical research institutes, and directors and managers from the public hospitals. For the quantitative survey, the research subjects were hospital managers who are working at public hospitals in different levels.

### 2.2. Sampling methods, data collection and analysis

#### 2.2.1. Document analysis

Position description (PD) for the management levels was voluntarily provided by four public general hospitals and individual members of Department of Health in Ninh Binh and Yen Bai provinces. Each province conveniently provide information from both a provincial and a district hospital. PD content analysis was conducted by three researchers. The list of tasks and job requirements as detailed in each of the PD were entered into an Excel spreadsheet. Content analysis was carried out to identify similarities in tasks and job requirements among management positions in hospitals.

#### 2.2.2. Competency identification

Identification of leadership and managerial competencies was conducted by the above three researchers. Based on a literature review, the relevant competency factors and items were identified. These competency factors and items were found to be useful for hospital managers to perform their tasks and job requirements.

#### 2.2.3. Workshop to validate identified competencies

A workshop was organized to reach a consensus on a leadership and managerial competency framework for different management levels such as departments of health, hospitals and preventive health centres. The workshop's participants were stakeholders who came from the Ministry of Health (9 participants), provincial departments of health (24 participants), medical universities and research institutes (25 participants) and public hospitals (43 participants). It is notable that fifty participants were involved in hospital managerial competency validation.. Out of those, 43 were from public hospitals and three were from Ministry of Health and four from medical universities and research institutes. A voting technique was used to reach the participants' agreement on these competencies.

#### 2.2.4. Quantitative survey for verification

After the workshop, a quantitative survey was carried out to verify the managerial competencies. This survey was conducted at public hospitals located in seven geographically representative provinces such as Ha Noi, Ho Chi Minh City, Ninh Binh, Khanh Hoa, Gia Lai and Dong Thap. In each province or city, a representative sample of provincial and district hospitals was selected. The total number of surveyed hospitals was 38. All hospital managers were invited to participate into data collection. In total, 891 hospital managers were involved in the survey. The groups of selected staff from Hanoi University of Public Health conducted the surveys. A self-reporting instrument was developed using Likert-scale of 5 levels from (1) very unimportant to (5) very important. It took about 45 minutes for the research subjects to complete the survey. The collected data were analysed using SPSS version 20. The exploratory factor analysis (EFA) to confirm the competency factors and items and Cronbach's Alpha to assess internal-consistency reliability of these competency items. In order to have sound factor analysis, several conditions must be met. There must be some coefficients of 0.3 and above appearing in the correlation matrix of the items. The Bartlett's Test of Sphericity was significant (< 0.05) [19]. Thus, it means that the correlation among items is sufficient to run the EFA. The Kaiser-Meyer-Oklin value Measuring Sample Adequacy must be of 0.6 and above [20]. This means that the sample size is sufficient and relevant enough to run EFA and show no serious multicollinearity. The items show good internal-consistency reliability if Cronbach's alpha is of 0.7 and above [21].

The study was approved by the Ethics Committee of Hanoi University of Public Health, Vietnam. The surveyed individuals all signed the informed consent form.

## 3. Results

This part will present the findings of the study. The tasks required for hospital managers will be presented in the first step of document analysis and shown in Table 1. The identified and validated leadership and managerial competencies will be presented in the second step of competency identification and the third step of workshopping to validate the identified competencies is also shown in Table 1. Finally, the competency confirmation will be presented in the fourth step of quantitative survey for verification and shown in Table 2.

**Table 1.** Key tasks and essential competencies by hospital managers.

*Tasks*	*Competencies*
Development of hospital vision, strategic direction and policies Maintenance and improvement of quality of service provision Promotion and development of organizational image and public relations Staff management and development Financial management Asset management Networking and liaison with stakeholders Investigation, monitoring and evaluation of hospital regulations Disaster control	Policy development and implementation Strategy development and orientation Plan making Human resource management Financial management Equipment and infrastructure management Risk and disaster management Quality management Investigation Supervision Monitoring and evaluation Ethics knowledge Information management Self-management

**Table 2.** Reliability of Hospital Management Competency.

*N0.*	*Competency Factors*	*Number of Items*	*Cronbach's alpha*	*Mean total score*
1.	Policy development and implementation	12	0.95	3.76
2.	Strategy development and orientation	06	0.97	3.67
3.	Plan making	09	0.97	3.69
4.	Human resource management	05	0.99	3.73
5.	Financial management	10	0.97	3.57
6.	Equipment and infrastructure management	07	0.99	3.60
7.	Risk and disaster management	02	0.94	3.64
8.	Quality management	04	0.90	3.73
9.	Investigation	04	0.95	3.54
10.	Supervision	05	0.98	3.59
11.	Monitoring and evaluation	06	0.97	3.47
12.	Ethics knowledge	04	0.98	3.81
13.	Information management	02	0.95	3.57
14.	Self-management	05	0.96	3.75

### 3.1. Document analysis

A total of 82 PDs for hospital management positions were collected and analysed, which brought about the identification of nine key tasks for hospital managers.

### 3.2. Competency identification

In total, 12 competency factors and 74 items were identified through the literature review. These 14 competency factors include: (1) policy development and implementation (12 items); (2) strategy development and orientation (6 items); (3) plan making (9 items); (4) human resource management (5 items); (5) financial management (10 items); (6) equipment and infrastructure management (7 items); (7) risk and disaster management (2 items); (8) quality management (4); (9) investigation (4 items); (10) supervision (5 items); (11) monitoring and evaluation (6 items); (12) ethics knowledge (4 items). These competency factors and items were found to be useful for hospital managers to perform their tasks and job requirements.

### 3.3. Workshop to validate identified competencies

In the workshop, all participants agreed on the identified leadership and managerial competencies. As well, there was consensus around adding two more competency factors and seven additional items. Hospital managers need to have competencies of (11) information management (2 items) and (14) self-management (5 items). These two competencies are really needed as the participants agree that “information” plays very important role in helping to make decisions in hospital performance improvement and “self-management” such as conflict resolution skills, teamwork, communication, meeting organization, and decision making are all useful competencies in hospital management. Overall, the total number of managerial competency factors and items was 14 and 81 respectively. The number of competencies is higher than the number of tasks due to the fact that several competencies can be used to complete one task. It is clear that the competencies of 1, 2 and 3 are really needed for managers to complete task 1. The competencies 1 and 3 are also useful for managers in fulfilling all other tasks as they are generally essential competencies. The competencies of 4, 5, 6 are useful for resource management which is obviously needed to complete the task 4, 5 and 6 respectively. The competencies of 7, 8 are relevant to tasks 9 and 2 respectively. The competencies 9, 10 and 11 are highly relevant to task 8. The competencies of 12, 13 and 14 are needed for all the tasks. Once again, these competencies were confirmed to be useful for hospital managers to complete their tasks and job requirements.

### 3.4. Quantitative survey for verification

The 81 items on the leadership and managerial competencies scale were used to put into EFA using SPSS. Prior to performing EFA, the appropriateness of data for factor analysis was evaluated. It was found that many coefficients of 0.3 and above appear in the correlation matrix. The Kaiser-Meyer-Oklin value was 0.98, exceeding the recommended value of 0.6 and the Bartlett's Test of Sphericity was significant, supporting the factoribility of the correlation matrix.

EFA showed the presence of 14 components with eigenvalues exceeding 1, explaining 56.6 per cent, 5.3 per cent, 3.4 per cent, 2.8 per cent, 2.4 per cent, 2.2 per cent, 1.9 per cent, 1.6 per cent, 1.5 per cent, 1.4 per cent, 1.3 per cent, 1.3 per cent, 1.2 per cent and 0.9 per cent of the variance respectively. An inspection of the screeplot showed a break after the 14^th^ component. It was decided to gain 14 components for further investigation. In order to interpret these 14 components, Varimax rotation was done. The rotated solution revealed the presence of structure, with 14 components showing a number of strong loadings (> 3).

The Cronbach's Alpha and the mean total scores for the management competency factors or subscales are presented in Table 1 below. The Cronbach's Alpha for all scales is at a very good level of reliability, over 0.9 (Table 2).

## 4. Discussion

In this study, PD analysis has been conducted to identify the key tasks that hospital managers have to complete. Based on the results of the PD analysis, the study confirmed nine key tasks and essential competencies required to perform these tasks effectively. The PD analysis has been applied in several international studies [4],[6],[9],[12],[22]. After being identified, the leadership and managerial competencies were confirmed through the workshop which was held with the participation of many stakeholders from different organizations such as Ministry of Health, hospitals, medical universities and health research institutes. This important step helps to validate the competencies, confirming their relevance to the hospital managers' tasks and job requirements, but it was not always implemented in other studies [12]. The results of the workshop were that the identified competencies derived from the PD analysis were confirmed and two more competencies of “self-management” and “information management” were agreed to be added in. These competencies play very important roles in helping hospital managers to cope with the issues arising from the new context in which decentralization, autonomous policies have been made. The final step to validate the agreed competencies used the statistical technique of EFA to analyse the survey data of 891 hospital managers. This kind of competency validation has been globally conducted [9],[22],[23]. In this survey, the response rate was 85% which is good given that response rates in studies of professionals using self-administered instruments are generally poor [24]. This may indicate that hospital managers are increasingly interested in hospital management and management development.

Overall, 14 leadership and managerial competency components with eighty one items were confirmed using EFA. These items revealed the high internal-consistency reliability as Cronbach's alpha > 0.9. This finding is similar to that of several studies [9],[12],[17],[22]. Other studies did not apply this technique for competency validation, despite many competencies being identified [7], and other studies applied only the qualitative methods [6].

Although hospital managers have been working at different levels, they shared the same competencies because they have done similar tasks and job requirements [18]. These competencies are: Policy development and implementation; strategy development and orientation; plan making; human resource management; financial management; equipment and infrastructure management; information management; risk and disaster management; self-management; quality management; investigation; supervision; monitoring and evaluation; and ethics knowledge. There have been arguments advanced that requirements for hospital managers in different levels are different [25], thus requiring different health workforce strategies [13]. This study contradicted these arguments by confirming that similar managerial competencies exist for hospital managers at all level. The perception that managers at different hospital levels (from central to district levels) share the same competencies has been documented [7],[26]–[28].

It is argued that competency is context dependent and developmental, and that required competency levels may need to vary accordingly [29]. Therefore, a generic competency-based education and training curriculum may be developed with specific strategies included to help managers translate the knowledge into practice in different contexts. In order to achieve this, different training approaches should be adopted such as problem-based training, coaching and mentoring.

Of these 14 leadership and managerial competencies identified for hospital managers, policy development and implementation; strategy development and orientation; plan making; human resource management; financial management; equipment and infrastructure management; information management; risk and disaster management; self-management; investigation; supervision; monitoring and evaluation; ethics knowledge have all been widely identified in the previous studies [6],[9],[22] while leading and quality management have not been clearly recognized. As mentioned in the introduction, healthcare has undergone significant changes since early 1990s in Vietnam. The introduction, implementation, supervision, monitoring and evaluation of large-scale changes, including major restructuring and adoption of service delivery models, especially implementation of quality improvement are the key responsibilities of hospital managers. Leading and managing these changes are very important for hospital managers [6]. In order to measure the quality of hospital performance, the Ministry of Health has issued 83 standards for hospital performance [30], which require the participation of all hospital managers to ensure the highest quality performance levels. Quality improvement is an innovation which plays a very important role in guaranteeing success in the challenging healthcare environment. However, the lack of interest shown by hospital managers in quality-improvement regimes in their hospitals has led to failings in this area. The failure is also caused by different perceptions held by managers and their staff towards quality improvement and its benefits, and by the lack of skills and experience in developing and implementing the change plan [6]. These require hospital managers to develop competence in quality management.

This study has several strengths. First, that the identification of leadership managerial competencies based on international literature was confirmed by hospital managers working in the real context. Second, that the quantitative survey was conducted using a large sample size of hospital managers at seven geographically representative regions. Third, the study applied an advanced statistical technique to validate competency model. Albeit, the study does have a limitation. Although being conducted in seven ecological regions, the study is not generalizable.

## 5. Conclusions

The results of the analysis confirmed the essential leadership and managerial competencies for public hospital managers in Vietnam. These competencies include: Policy development and implementation; strategy development and orientation; plan making; human resource management; financial management; equipment and infrastructure management; information management; risk and disaster management; self-management; quality management; investigation; supervision; monitoring and evaluation; ethics knowledge. These are necessary competencies if managers are to fulfill their tasks effectively and will be used as a basis to develop the competency-based training for the current management taskforce and preparing future hospital managers. This kind of the study was limited to representative provinces in seven geographical regions. Thus, it should be further studied to gain the overall and clearer picture of leadership and managerial competencies for hospital public managers.
